# Curvature dependence of BAR protein membrane association and dissociation kinetics

**DOI:** 10.1038/s41598-022-11221-9

**Published:** 2022-05-10

**Authors:** Rui Jin, Rui Cao, Tobias Baumgart

**Affiliations:** 1 Department of Chemistry, University of Pennsylvania, Philadelphia, PA USA; 2 Division of Biostatistics, University of Minnesota, Minneapolis, MN USA

**Keywords:** Membrane biophysics, Membrane structure and assembly, Molecular biophysics

## Abstract

BAR (Bin/Amphiphysin/Rvs) domain containing proteins function as lipid bilayer benders and curvature sensors, and they contribute to membrane shaping involved in cell signaling and metabolism. The mechanism for their membrane shape sensing has been investigated by both equilibrium binding and kinetic studies. In prior research, stopped-flow spectroscopy has been used to deduce a positive dependence on membrane curvature for the binding rate constant, *k*_*on*_, of a BAR protein called endophilin. However, the impact of bulk diffusion of endophilin, on the kinetic binding parameters has not been thoroughly considered. Employing similar methods, and using lipid vesicles of multiple sizes, we obtained a linear dependence of *k*_*on*_ on vesicle curvature. However, we found that the observed relation can be explained without considering the local curvature sensing ability of endophilin in the membrane association process. In contrast, the diffusion-independent unbinding rate constant (*k*_*off*_) obtained from stopped-flow measurements shows a negative dependence on membrane curvature, which is controlled/mediated by endophilin-membrane interactions. This latter dependency, in addition to protein–protein interactions on the membrane, explains the selective binding of BAR proteins to highly curved membranes in equilibrium binding experiments.

## Introduction

The Bin-Amphiphysin-Rvs (BAR) domain protein superfamily is a large group of proteins that are involved in bio-membrane shape changes^1,2^. These proteins are known to form crescent-shaped dimers^3,4^. BAR proteins are involved in numerous membrane trafficking pathways and events such as the formation of membrane buds, and the scission of vesicles from donor membranes^5^. For example, FCHO proteins containing F-BAR domains are involved in the nucleation of clathrin-coated membrane pits in clathrin-mediated endocytosis^6^. N-BAR proteins such as endophilin and amphiphysin, contain an N-terminal amphipathic helix which facilitates binding to the neck of clathrin-coated vesicles to facilitate downstream membrane scission^7,8^. Endophilin is also involved in a fast endophilin-mediated endocytosis mechanism which is clathrin-independent^9,10^.

In vivo and in vitro studies have been carried out to investigate the relation between membrane curvature and BAR protein binding^11–16^. BAR proteins inherently sense curvature: they have a higher binding affinity to bent membranes compared to planar membranes^17–19^. At sufficiently high protein densities, they reshape the membrane into bent morphologies such as tubules or membrane buds^17,18,20,21^. These BAR proteins can form tip-to-tip oligomers on lipid membranes and lattice structures on membrane tubules, which may contribute to membrane binding and curvature generation^22–25^. The curved BAR domain, the helix insertion of N-BAR proteins, as well as linear protein oligomerization on membranes, have all been proposed to contribute to BAR protein membrane curvature sensing and generation^26–28^. The membrane binding of N-BAR proteins is known to be facilitated by the N-terminal helix (H0) insertion into the lipid headgroup region^12,27,29,30^. Experiments with tubules pulled from (almost) planar lipid membranes covered by N-BAR proteins revealed that these proteins preferentially bind to bent membranes^17,18,23,31^. Both small and large unilamellar vesicles (SUVs, diameters < 30 nm; LUVs, diameter > 100 nm) as well, have been used to confirm the preferred binding of N-BAR proteins from the aqueous environment to smaller vesicles compared to larger ones^11,12^.

Several steps are involved in the membrane binding process such as protein association, dissociation and filament formation of BAR proteins mediated by H0 interactions^21,22,32,33^. While equilibrium studies cannot distinguish which binding step is membrane curvature related, kinetic studies shed light on this question since they enable, in principle, the measurement of rate constants for each step^34,35^.

Kinetic measurements on protein-membrane interaction have been realized with a variety of techniques^36,37^. Single molecule experiments track the retention time of membrane-bound proteins to obtain protein dissociation rates^38^. In surface plasmon resonance (SPR) experiments^39,40^ and quartz crystal microbalance studies^41,42^, constant flows of protein solution and protein-free buffer are applied onto membrane coated sensors, enabling the quantification of protein association and dissociation kinetics. Techniques incorporating waveguide components, such as dual polarization interferometry^43,44^, plasmon waveguide resonance^45,46^ and optical waveguide light mode spectroscopy^47,48^, have also been implemented in protein–membrane interaction kinetics. However, the above techniques have mostly been applied in protein binding studies onto planar lipid membranes with a supported lipid bilayer formed on the sensor surface. To incorporate SUVs or LUVs of different curvatures in these experiments, an additional tethering step is needed^49,50^.

Stopped-flow mixing combined with Foerster resonance energy transfer (FRET) to detect membrane binding, is a versatile technique to obtain both the binding and the unbinding kinetics of proteins onto vesicles prepared to have different sizes^51–53^. Moreover, fluorescent LUVs can be directly used without further modification, which allows for quick repetitions of the measurements for statistical results and investigation of binding kinetics with varied conditions. Time resolution of stopped-flow can be on the order of less than one millisecond for tracking rapid interactions between proteins and lipid vesicles^54,55^. The encounter and interaction of membranes and proteins is influenced by the diffusion behaviors of the two particles in the mixture^56^. In extreme circumstances, the binding rate constant can be completely diffusion-controlled^57,58^. In these situations, the on-rate is not related to the affinity of the protein-membrane interaction.

Stopped-flow spectroscopy with FRET readout has previously been used to probe membrane binding kinetics of endophilin^34,35^. A positive dependence of the binding rate constant on membrane curvature was observed comparing LUVs of two different sizes^35^. However, the question if this represents a molecular, i.e. local, curvature sensing driven observation, or if this can be attributed to diffusion-controlled protein binding (which would not be affected by local membrane curvature), has not been asked. Furthermore, the limited range of different vesicle sizes did not allow to assess the functional relationship between vesicle radii and binding kinetics.

In this contribution, we applied stopped-flow mixing combined with FRET measurements to examine the binding kinetics of the endophilin N-BAR domain to LUVs of a range of different sizes. We find that the binding rate constant shows a linear dependence on membrane curvature. We then show that the observed curvature dependence is explained by a diffusion-controlled binding process, which cannot be attributed to molecular membrane curvature sensing during the association step. In contrast, the unbinding rate is observed to decrease with increasing membrane curvature.

## Materials and methods

### Materials

The lipids 1-palmitoyl-2-oleoyl-sn-glycero-3-phosphocholine (POPC), 1,2-dioleoyl-*sn*-glycero-3-phospho-l-serine (DOPS) and 1,2-dipalmitoyl-*sn*-glycero-3-phosphoethanolamine-*N*-(7-nitro-2–1,3-benzoxadiazol-4-yl) (16:0 NBD PE) were obtained from Avanti Polar Lipids (Alabaster, AL). The dye Pacific Blue™ (PB) C5-maleimide was obtained from Invitrogen/Life Technologies (Grand Island, NY). 4-(2-hydroxyethyl)-1-piperazineethanesulfonic acid (HEPES) was from SIGMA-ALDRICH^®^; sodium chloride (NaCl), tris (2-carboxyethyl) phosphine (TCEP), Coomassie plus (Bradford) protein assay reagent and bovine serum albumin (BSA) standards were from Thermo Fisher Scientific. All commercial reagents were used without further purification.

### Protein preparation

A plasmid encoding rat endophilin A1, kindly provided by P. De Camilli, served as the template to generate endophilin N-BAR (ENB) C108A L187C (1–247) mutant, which was verified by DNA sequencing. The L187C mutation was created at the tip region of ENB for PB labeling that served as the donor in the PB/NBD FRET pair.

The GST fusion protein was purified from bacterial lysates (BL21(DE3) RIL CodonPlus, Stratagene) using glutathione affinity. The GST moiety was cleaved by addition of PreScission protease with 1:50 enzyme to protein molar ratio. The mixture was then shaken at 4 °C for 8 h to achieve complete cleavage. The GST tag and ENB were separated by ion exchange with a linear NaCl gradient (NaCl concentration increased from 150 to 400 mM with 50 mM Tris to maintain pH 8) in a HisTrap Q HP anion exchange chromatography column (GE healthcare). The products were then further purified with size exclusion chromatography (SEC) (Superdex200, GE Healthcare) in 20 mM HEPES, 150 mM NaCl, 1 mM TCEP, pH 7.4 solution (HN150T buffer). Protein identity and purity was assessed by SDS-PAGE after each purification step. The products were concentrated in HN150T buffer, flash-frozen via liquid nitrogen and stored at −80 °C. Before each set of measurements with thawed samples, we applied ultracentrifugation to remove potential aggregates. Concentrations were determined by Bradford assay using BSA as a standard. Concentrations indicated refer to the ENB concentration in terms of monomeric units. Labeling with PB at position 187 was accomplished by adding a fivefold excess of maleimide dye reagent to the ENB solution for reaction at 4 °C. Reactions were quenched with excess dithiothreitol (DTT), and excess dye reagent was removed via three 5-ml HiTrap desalting columns (GE Healthcare) connected in series. The final labeling efficiency varied between 10 and 70%. For further kinetic studies, ENB was diluted in an HN50T buffer with lower ionic strength compared to HN150T. (HN50T: 20 mM HEPES, 50 mM NaCl, 1 mM TCEP, pH 7.4 solution).

### Preparation of large unilamellar vesicles (LUVs)

Chloroform solutions of 70 mol% DOPC, 25 mol% DOPS and 5 mol% 16:0 NBD PE lipid stock were generated in a round-bottom flask. The lipid solution was gently evaporated under a nitrogen flow and then transferred to vacuum for 2 h to completely remove the solvent. Lipids were hydrated to obtain solutions of 1–2 mM lipid concentration in a buffer consisting of 20 mM HEPES, 50 mM NaCl, 1 mM TCEP, at pH 7.4 (HN50T buffer). Lipid dispersions were extruded 21 times through single polycarbonate membranes (Whatman/GE Healthcare) with pore sizes of 50, 100, 200, 400 and 800 nm. To help generate small LUVs before extrusion through 50 nm and 100 nm membranes, the LUV dispersions underwent two freeze–thaw cycles, in which the samples were flash-frozen in liquid nitrogen and then sonicated for 5–10 min. The size distribution of LUVs in each sample was determined by dynamic light scattering (Zetasizer nano, Malvern). We caution that important recent experiments have shown that conditions of low fractions of negatively charged lipids as well as high ionic strength (such as 1× PBS buffer) can lead to the generation multilamellar vesicles by the method of vesicle extrusion^59^. The fraction of negatively charged lipids (25 mol% DOPS + 5 mol% NBD PE = 30 mol%) is significantly larger, and ionic strength used in our experiments significantly smaller, than that of vesicles and solution conditions considered in those experiments and we therefore consider our vesicles to be unilamellar.

### Stopped-flow experiments for ENB binding

Measurements were carried out at 22 °C with a Kintek stopped-flow spectrometer (AutoSF120) using excitation at 402 nm and collecting emission after passage through a 440/40 nm filter, corresponding to PB’s excitation and emission wavelengths. 200–500 µl of the two solutions were loaded to separate sample injectors and 20 µl of each were mixed each time. With sample discarding, 10–25 traces were collected and averaged for each condition. The averaged trace was fit to a single-exponential decay to obtain the corresponding kinetic parameter *k*_*obs*_.

**Figure 1:**
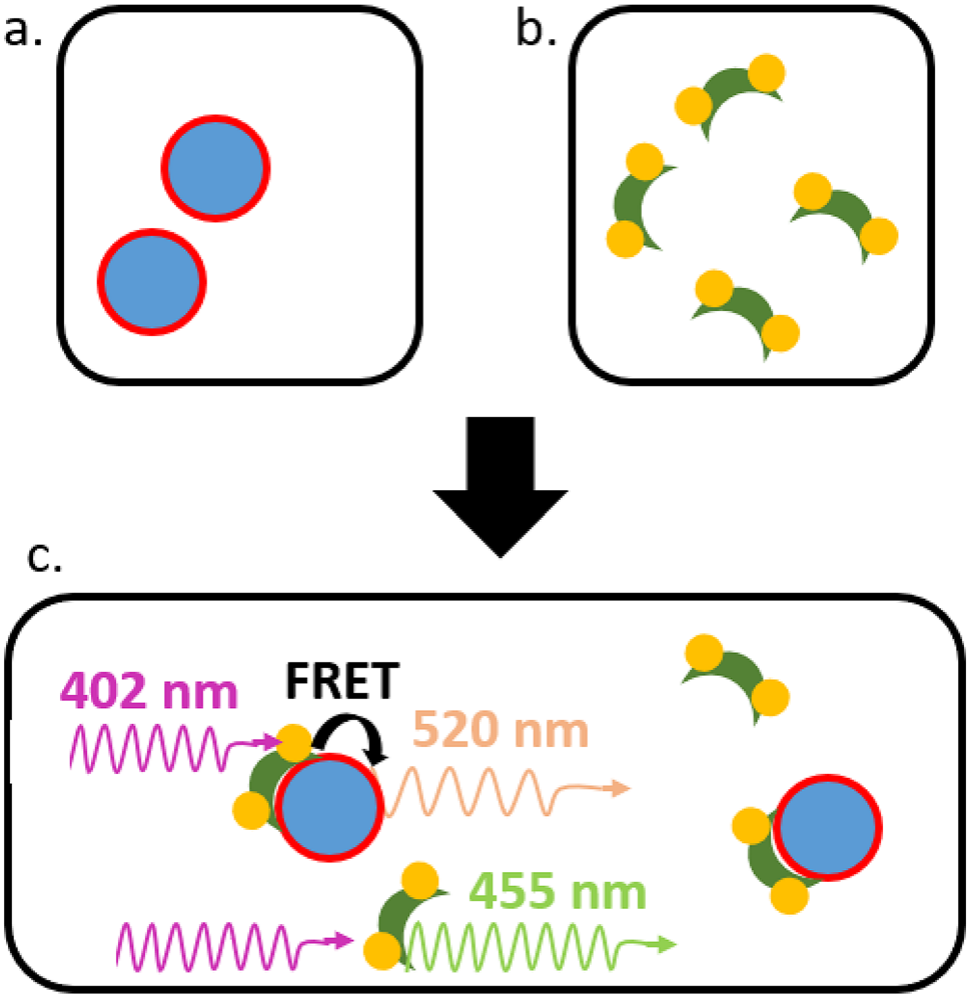
Stopped-flow experiment to obtain *k*_*on*_ and *k*_*off*_ of ENB with lipid membranes. Pacific blue (PB)-labeled ENB **(a)** is mixed with NBD-containing LUVs **(b)** of different concentrations. **(c)** FRET between two fluorophores causes the emission intensity of PB (EX: 402 nm), the donor, to decrease (collected with 440/40 nm filter) when ENB is membrane bound (top), relative to PB dyes on ENB that is not bound to membranes (bottom). Such decrease reflects the binding of ENB onto the LUV surface. The relation between obtained binding rates *k*_*obs*_ and the lipid concentration can be used to obtain *k*_*on*_ and *k*_*off*_.

In a 1:1 volume ratio, 1 µM PB-labeled ENB (diluted in HN50T) was mixed with NBD-containing LUVs of total lipid concentration 0.25, 0.50, 0.75 or 1.00 mM prepared in the same HN50T buffer (Fig. 1). LUVs of different average sizes were applied in the same experiments. After mixing, the concentration of ENB and lipids each decreased by a half (e.g. [ENB] = 0.5 µM). The fluorescence was collected for 0.1 s with a 440/40 nm fluorescence filter (Edmund Optics). Within this time scale, we assumed the association of ENB with the lipid membrane to follow a simple binding-unbinding model without forming complex oligomers on the membrane surface. The concentration changes of bound protein (BP) and free protein in the solution (FP) followed the relation:

1 $$\frac{d\left[BP\right]}{dt}={k}_{on}\left[FP\right]\left[lipid\right]-{k}_{off}[BP],$$

where *k*_*on*_ is the association rate constant and *k*_*off*_ is the dissociation rate constant. When the lipid concentration is much larger than the ENB concentration,

2 $$\left[BP\right]={C}_{1}{e}^{{-k}_{obs}t}+{C}_{2}$$

3 $${k}_{obs}={k}_{on}\left[lipid\right]+{k}_{off}$$

Here, *C*_*1*_ = -*C*_*2*_ = -*k*_*on*_ [P]/*k*_*obs*_ with [P] = [BP] + [FP] = 0.5 µM. Since FRET between PB and NBD caused the emission intensity of PB to decrease as a consequence of ENB binding to the NBD LUVs, *k*_*obs*_ was obtained from the traces collected by the stopped-flow spectrometer. The linear fitting of *k*_*obs*_ and the lipid concentration gave the slope as *k*_*on*_ and the intercept on the *k*_*obs*_ axis as *k*_*off*_.

## Results

To investigate if the binding kinetics of endophilin N-BAR (ENB) onto lipid membranes is dependent on membrane curvature, we performed stopped-flow experiments to measure the binding and unbinding constants, *k*_*on*_ and *k*_*off*_. NBD-labeled vesicles of different sizes were prepared via LUV extrusion through polycarbonate membranes with different pore diameters and their average diameters were measured by dynamic light scattering (DLS). For LUVs of each size, we tracked ENB binding in the stopped-flow experiments via the decrease in fluorescence of PB labels covalently coupled to the tip region of ENB (Fig. 1).

**Figure 2:**
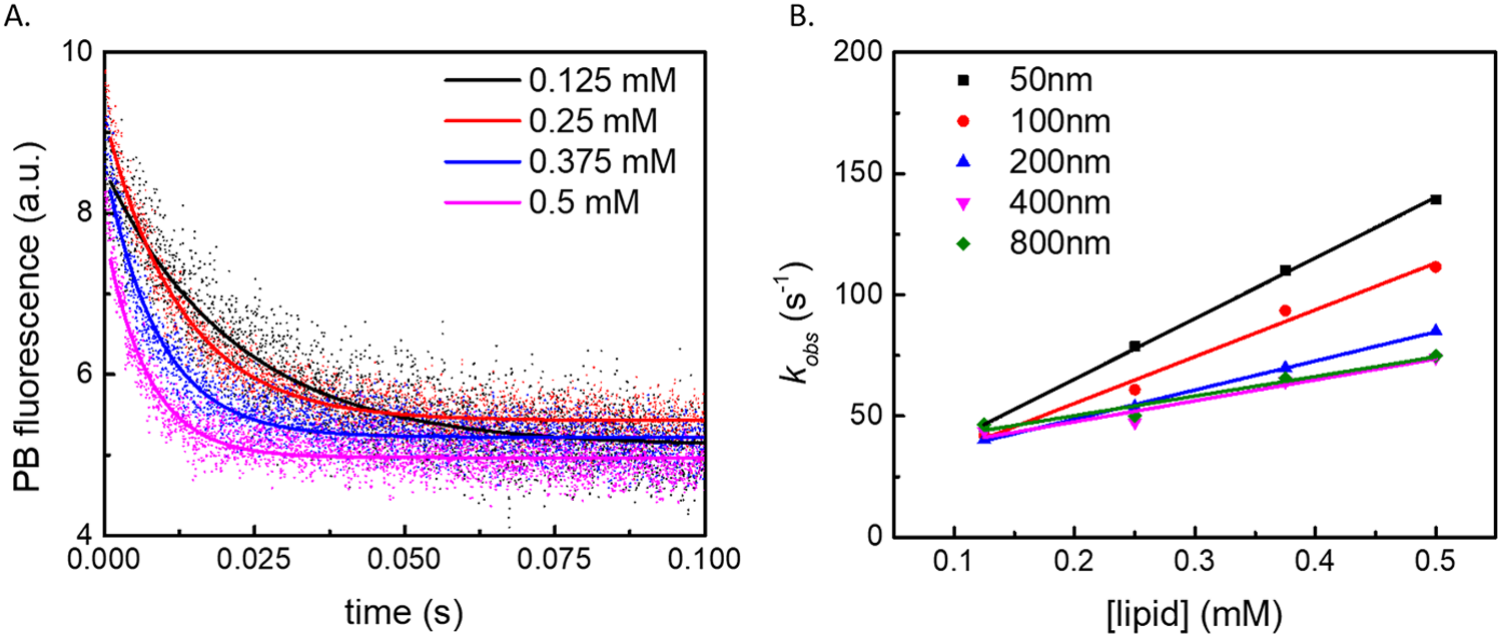
*k*_*obs*_ values for ENB binding experiments are different for LUVs of different sizes. **(A)** The PB fluorescence change after mixing 1 µM ENB with 50 nm LUVs of different concentrations. The signal decay rate increases as the concentration of LUVs increases as predicted by Eq. (2). The data shown resulted from averaging 10–25 consecutive measurements, as described in the “Materials and methods” section. **(B)** The relation between *k*_obs_ and lipid concentration for LUV samples of different sizes obtained through extrusion through filters with pore sizes indicated in the legend. *k*_obs_ and [lipid] follow a linear relation as predicted by Eq. (3). The slope, which denotes *k*_on_, increases as the size of the LUVs decreases. Error bars are the standard errors from the exponential fitting in **(A)**, which are smaller than the symbol size. **(B)** shows the results from a single vesicle preparation.

1 µM PB-labeled ENB was mixed at a 1:1 volume ratio with lipid vesicles at lipid concentrations of 0.25 mM, 0.50 mM, 0.75 mM or 1.0 mM. Hence, the concentration of both ENB and lipids decreased by half due to mixing. Figure 2A shows time traces of the fluorescence signals collected for LUVs extruded through a 50 nm filter. The figure shows that the rate of protein binding increases when the lipid concentration is increased, as expected. The fluorescence signal collected through a 440/40 nm filter corresponds to the PB emission from free protein in solution, therefore the fluorescence signal decay is consistent with a decrease of the solution concentration of unbound protein and a corresponding increase of protein binding to the lipid vesicles. By fitting the signal change to Eq. (2), we were able to determine the observed binding rate constant *k*_*obs*_ as a function of different lipid concentrations (Fig. 2B). As implied by Eq. (3), *k*_*on*_ and *k*_*off*_ can be obtained by linear fitting of the lipid concentration dependence of *k*_*obs*_ shown in Fig. 2B, with *k*_*on*_ corresponding to the slope and *k*_*off*_ to the intercept.

Equations (2) and (3) can be used to interpret our experimental findings because the lipid concentration was much larger than the protein concentration (250–1000 fold). These concentrations were chosen so that endophilin proteins did not need to compete with bound molecules due to a limited number of binding sites. Under the following assumptions: (1) all available ENB binds to the membrane surface, (2) ENB dimers cover an area of roughly 3 × 12 nm^2^ based on its crystal structure^14^ and (3) the average lipid headgroup area is 0.65 nm^2^ per lipid^60–62^, the total membrane surface area was 2–9 times of the total area needed for *all* the ENB to bind to LUVs. Thus, in Eq. (1) we can regard the total lipid concentration available for binding as approximately constant during the binding process and Eqs. (2) and (3) are derived based on this condition.

**Figure 3:**
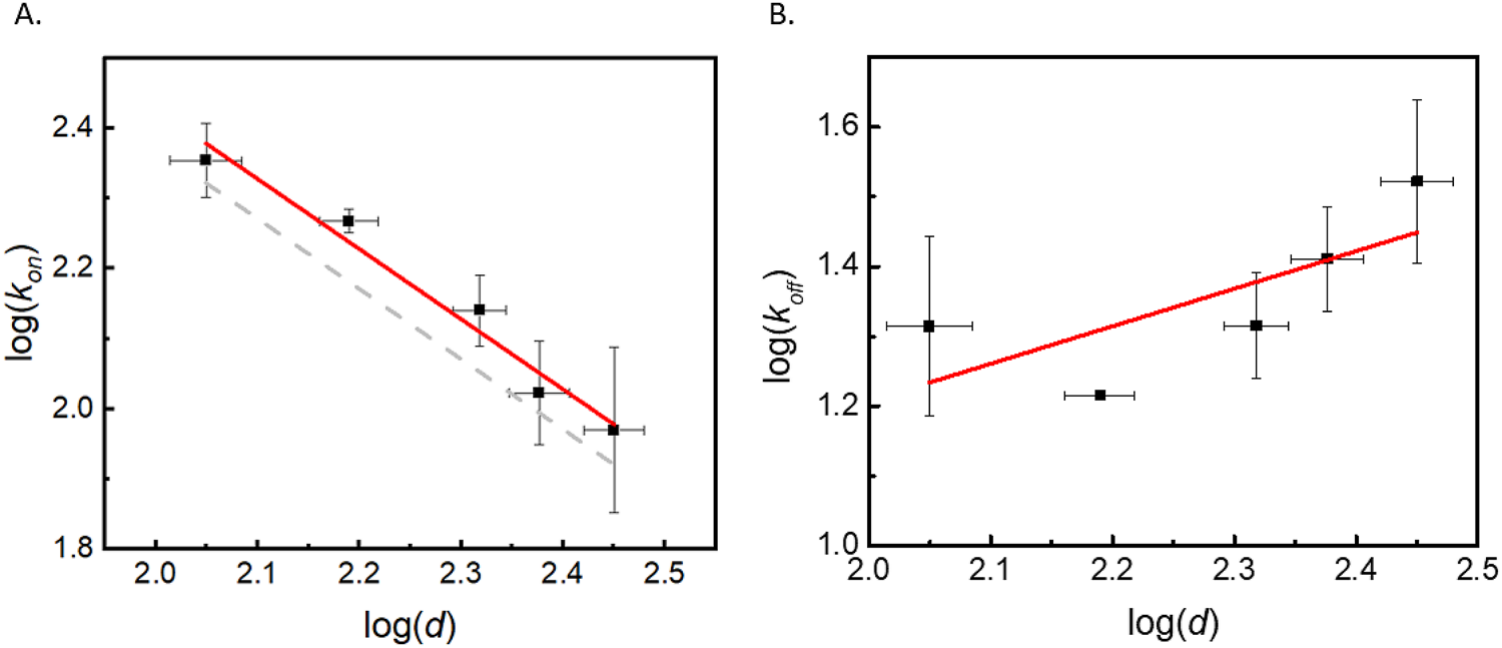
Relation of *k*_*on*_ and *k*_*off*_ with the average diameter (*d*) of the LUVs. In both panels, *d* was determined through dynamic light scattering measurements. **(A)** The relation between log(*k*_*on*_) and log(*d*), shows a negative linear dependence. The slope of the linear fitting (red solid trend line) is −1.00 ± 0.10. The grey dashed line represents a theoretical estimate according to diffusion constant estimates and Eq. (8). **(B)** log(*k*_*off*_) shows a positive dependence on log(*d*). The slope of the linear fit is 0.54 ± 0.28. Error bars of log(*d*) are from the size distribution of each sample determined by DLS. *k*_*on*_ and *k*_*off*_ were obtained by averaging the fitting results obtained via Eq. (3) from three different vesicle preparations, the results for a representative single one of these are shown in Fig. 2B. The error bars for log(*k*_*on*_) and log(*k*_*off*_) are the standard deviations from three different vesicle preparations.

Figure 3 presents *k*_*on*_ and *k*_*off*_ obtained from the linear fitting via Eq. (3) of data such as shown in Fig. 2B for vesicles of different sizes. As the curvature decreases (radius increases), the binding rate constant *k*_*on*_ decreases and the unbinding rate *k*_*off*_ increases. For *k*_*on*_, the calculated value is linearly dependent on the membrane curvature (1/*R*_LUV_), given a slope of the double-logarithmic plot equal to 1 within experimental uncertainties (Fig. 3A). Similar observations have been made in other studies of protein/membrane binding kinetics, and such observations have led the authors to suggest that the protein binding is dependent upon membrane curvature^35,63^. However, it is important to note that by varying the vesicle size, the number density of vesicles also varies (at fixed lipid concentration). Meanwhile, the LUV size influences the collision frequency between peripheral proteins and LUVs, and this is an important factor in determining the binding rate.

In principle, there are (at least) two mechanisms involved in protein binding to the LUV surface: the first step is the diffusion of protein to the membrane surface following a concentration gradient (under stopped-flow conditions) in the solution with a rate constant *k*_*diff*_. The second step involves reorientation of the protein and the insertion of the H_0_ and H_1i_ helices into the hydrophobic core of the lipid bilayer to complete the binding with rate constant *k*_*r*_. We can consider these two rate constants as a diffusion rate, and a “reaction rate”, respectively. According to Smoluchowski’s theory^64^, we can relate the on-rate as defined by Eq. (1) to the rate constants of the two consecutive steps just described:

4 $${k}_{on}=\frac{{k}_{diff} {k}_{r}}{{k}_{diff}+{k}_{r}}$$

If the first step is significantly slower than the second one, the binding process is considered to be diffusion-controlled and thus *k*_*on*_ can be approximated by *k*_*diff*_, which is related to the collision frequency (*k*_*coll*_) between LUV and an ENB dimer, as we explain below. According to Smoluchowsky theory, the collision rate is only dependent on the diffusion properties of the two interacting particles^64^:

5 $${k}_{coll}=4\pi {N}_{A}({R}_{ENB}+{R}_{LUV})({D}_{ENB}+{D}_{LUV})$$

Here, *N*_*A*_ is the Avogadro constant, *R*_*ENB*_ and *R*_*LUV*_ correspond to the radius of ENB and LUV, respectively, and *D*_*ENB*_ and *D*_*LUV*_ are the diffusion constants of the two particles. Since the size of LUVs is much larger than that of an ENB dimer and the latter diffuses much faster than LUVs, Eq. (5) can be simplified as^58^:

6 $${k}_{coll}=4\pi {N}_{A}{R}_{LUV}{D}_{ENB}$$

Therefore, increasing surface area of LUVs will lead to a larger collision frequency with ENBs, and thus the binding rate is expected to be linearly dependent on LUV radius *R*_*LUV*_ if the concentrations of LUVs are the same, even in the absence of any molecular effects of membrane curvature on protein binding.

When the concentration of lipids, [lipid], is kept constant, the LUV concentration [LUV] is dependent on the number of lipids per LUV, which can be estimated based on the headgroup area of one lipid molecule (*A*_*lipid*_) and the surface area of the LUV bilayer membrane:

7 $$[LUV]=\frac{{A}_{lipid}}{8\pi {R}_{LUV}^{2}}\left[lipid\right]$$

If we assume that each collision leads to a successful binding event, then the diffusion-controlled binding constant can be estimated via Eqs. (6) and (7):

8 $${{k}_{on}\cong k}_{diff}={k}_{coll}\frac{[LUV]}{[lipid]}= \frac{{N}_{A}{D}_{ENB}{A}_{lipid}}{2}\frac{1}{{R}_{LUV}}$$

The results imply that if the binding process is diffusion-controlled, no matter if membrane curvature affects *k*_*r*_ (such as the rate of protein helix insertion into the lipid bilayer), we expect *k*_*on*_ to be linearly dependent upon the reciprocal of *R*_*LUV*_, consistent with the experimentally observed relationship (Fig. 3A).

To confirm that the relation of *k*_*on*_ and *R*_*LUV*_ in Fig. 3A can be explained solely by a diffusion-limited reaction, we estimated *k*_*diff*_ based on Eq. (8) and compared it to *k*_*on*_ obtained from the stopped-flow experiments. *D*_*ENB*_ is estimated via Hydropro^65^ to be 6 × 10^–7^ cm^2^/s at *T* = 22 °C. The headgroup area of PC lipids, 0.65 nm^2^, is used for *A*_*lipid*_^62^. The vesicles filtered through 50 nm pores have an average radius of 56 nm. *k*_*diff*_ can be estimated to be 2.1 × 10^5^ s^−1^ M^−1^ by Eq. (8). The value of *k*_*on*_ determined by the stopped-flow, based on Eq. (8), is (2.3 ± 0.3) × 10^5^ s^−1^ M^−1^. The two values are quite close, which implies that ENB binding kinetics is diffusion-controlled. The grey dashed line in Fig. 3A shows the dependency of k_on_ on vesicle size based on the Hydropro estimate for the diffusion constant and Eq. (8).

We note that, interestingly, the experimentally determined values for *k*_on_ are slightly larger than (although within uncertainties of) the diffusion-limited rate constant *k*_diff_ determined for ENB (see Fig. 3A). In principle, the diffusion limit is the fastest rate that the binding reaction can obtain. However, so far, the electrostatic interaction between the DOPS-containing vesicle and ENB protein has not yet been considered. Since the concave face of the ENB dimer is positively charged^14^, the electrostatic attraction to the negatively charged DOPS in the LUV membrane contributes a slightly faster approach rate of ENB to the LUV surface^66,67^. Due to the screening effect of 50 mM NaCl in the solution, the electrostatic interaction only plays a role at short separation distances^68,69^. That may explain why we observe a slightly larger but not significantly different *k*_*on*_ compared to the estimated *k*_*diff*_.

In summary, we find that the relation between *k*_*on*_ and membrane curvature obtained from the binding kinetic studies is not related to a molecular curvature-dependent binding of ENB. Instead, it is determined by the diffusion behavior of ENBs and lipid vesicles. We do, however, observe that *k*_*off*_ decreases as the membrane curvature increases, which implies that ENB binds more tightly to lipid membranes of higher curvature. That may explain why ENB binds specifically to highly curved biomembranes such as the neck of buds in clathrin-mediated endocytosis^70^.

## Discussion

LUVs of different diameters have been widely applied to study the binding affinities and kinetics of peripheral proteins, including BAR proteins^34,35,63,71,72^. However, our findings show that caution is required in the interpretation of the relation between membrane curvature and binding parameters. The diffusion-controlled binding rate constant, *k*_*on*_, shows a linear dependence on membrane curvature^35^, which may not relate to molecular curvature sensing behavior. With fixed lipid concentration, the increase of *k*_*on*_ for smaller LUVs results from a combination of a varied conversion factor from vesicle concentration to lipid concentration (Eq. 7) and decreased protein-vesicle collision frequency for increased vesicle cross-section area^57,58^.

The curvature sensing property of BAR proteins has usually been quantified under conditions of a binding equilibrium. Sedimentation assays show more protein binding for LUV batches of smaller average size^11^. The single liposome curvature (SLiC) assay enables the quantification of protein area-density on the membrane of single LUVs whose diameters span 50–800 nm^12,29,30,73^. The relation between protein concentration in bulk solution and its density on LUVs of the same size can be fitted by the Langmuir adsorption equation to obtain *K*_*D*_, the apparent dissociation constant and *B*_*max*_, the surface density of membrane-bound molecules at saturation^12^. In our kinetic study, *B*_*max*_ was not called for in the data analysis since we used conditions where lipid concentration was much larger than the protein concentration so that any protein competition in the binding to the same binding site was negligible. Moreover, *K*_*D*_ obtained through measurements at binding equilibrium does not correspond to the ratio of *k*_*off*_ and *k*_*on*_ obtained in our kinetic measurement. As discussed above, *k*_*on*_ obtained in our study is controlled by the protein flux along its concentration gradient created by the depletion of proteins in the aqueous phase by their binding to the LUV surface. However, when the binding and unbinding of proteins reaches equilibrium, the protein concentration around lipid vesicles is homogenous and equal to the bulk concentration. Thus, *k*_*on*_ at equilibrium is not diffusion-controlled. We can further ask if the equilibrium *k*_*on*_ (non-diffusion-controlled) can be calculated by the product of equilibrium *K*_*D*_ and *k*_*off*_ obtained in the stopped-flow study. In the situation of BAR proteins, this simple approach is not warranted since the apparent *k*_*off*_ at equilibrium with high binding density can be influenced by the formation of linear protein oligomers and networks on the membrane surface^24,25,32,33^.

Since BAR proteins can form linear aggregates on the membrane surface and thus the apparent unbinding rate measured is lower compared to the situation when oligomerization is not involved^21,22,32,74^, the interaction between the protein and the membrane can be better described by a (minimally) two-step mechanism. Here, the first step is the exchange between free protein in the solution and bound proteins on the membrane, a process which is dominated by the kinetic parameters *k*_*on*_ and *k*_*off*_. The second step is the formation of protein oligomers on the membrane surface^34^. Thus the dissociation rate measured at equilibrium is a combined result of both the protein-membrane interaction and protein–protein interactions on the membrane^21,74^. Even though a curvature dependence of *K*_*D*_ for BAR proteins has been observed^12^, it remains difficult to distinguish if the main contribution to this dependence is through a protein-membrane interaction that is curvature-dependent, or if the dominant effect is that protein oligomerization is curvature-sensitive. Furthermore, we note that in the biological context, membrane recruitment of endophilin involves protein–protein interactions such as with adaptor proteins lamellipodin^75^ and CIN85^76^.

In our experiments, we used the intercept of a linear fit of the experimental relationship between *k*_*obs*_ and the lipid concentration to obtain *k*_*off*_. The unbinding rate constant *k*_*off*_ decreases with increasing membrane curvature. That is, endophilin binds more tightly to membranes with higher curvature. Since our stopped-flow experiments were carried out within a 0.1 s time scale and achieved low protein density on the membrane, the impact of oligomerization likely was smaller than that in equilibrium binding studies where LUVs were incubated with relatively high protein to lipid ratio for several minutes and even up to an hour^11,12^. Our results imply that the dissociation rate constant is dependent on membrane curvature, contributing to the curvature sensing function of BAR proteins. This dependency can be explained by that fact that more hydrophobic defects can be found on highly curved membranes and these contribute to more stable membrane insertion of the H0 helices of BAR proteins (and extra Hi1 helix insertion of endophilin)^12,27,77,78^.

## Conclusion

The present study presents stopped-flow experiments on the binding kinetics of endophilin N-BAR on lipid vesicles of varied membrane curvature. The data analysis implies that the observed relation between the vesicle size and the binding rate constant *k*_*on*_ is caused by the diffusion-controlled encounter of proteins and lipid vesicles and thus the measured *k*_*on*_ is not affected by molecular curvature sensing behavior. In contrast, the obtained dissociation rate constant *k*_*off*_ decreases with increased membrane curvature, supporting the curvature sensing property of BAR proteins.
